# Macrolide resistance in *Mycobacterium abscessus*: current insights and future perspectives

**DOI:** 10.1093/jacamr/dlaf047

**Published:** 2025-04-02

**Authors:** Victoria L Nguyen, Kelly L Eick, Mingyu Gan, Taryn A Miner, Anne E Friedland, Allison F Carey, Kenneth N Olivier, Qingyun Liu

**Affiliations:** Department of Genetics, University of North Carolina at Chapel Hill, Chapel Hill, NC 27599, USA; Department of Genetics, University of North Carolina at Chapel Hill, Chapel Hill, NC 27599, USA; Department of Genetics, University of North Carolina at Chapel Hill, Chapel Hill, NC 27599, USA; Department of Genetics, University of North Carolina at Chapel Hill, Chapel Hill, NC 27599, USA; Division of Infectious Diseases, University of North Carolina School of Medicine, Chapel Hill, NC, USA; Department of Pathology, University of Utah, Salt Lake City, UT 84112, USA; Marsico Lung Institute, University of North Carolina at Chapel Hill, Chapel Hill, NC, USA; Department of Genetics, University of North Carolina at Chapel Hill, Chapel Hill, NC 27599, USA; Department of Microbiology and Immunology, University of North Carolina at Chapel Hill, Chapel Hill, NC 27599, USA

## Abstract

*Mycobacterium abscessus* (MAB) is a rapidly growing, non-tuberculous mycobacterium that has emerged as a significant pathogen in both pulmonary and extrapulmonary infections. It is rising in prevalence, especially among individuals with underlying lung conditions such as cystic fibrosis and chronic obstructive pulmonary disease, highlighting its growing clinical importance. The treatment of MAB infections is notoriously challenging due to intrinsic resistance to many antibiotics and low cure rates, typically <50%. Macrolides are a cornerstone in the treatment of MAB infections because regimens that include effective macrolide therapy are associated with higher cure rates. However, MAB possesses intrinsic and acquired drug resistance mechanisms against macrolides, complicating drug susceptibility testing and selection of highly effective treatment regimens. This review aims to provide a summary of the current understanding of macrolide resistance mechanisms in MAB. We explored the epidemiology of resistance in different countries and the molecular mechanisms involved. We have highlighted the variability in sensitivity of existing markers to predict phenotypic macrolide drug resistance across different countries, suggesting the involvement of unknown resistance mechanisms. By synthesizing current knowledge and identifying gaps in the literature, this review seeks to inform clinical practice and guide future research efforts in the fight against MAB drug resistance.

## Introduction

*Mycobacterium abscessus* (MAB), characterized as a rapidly growing non-tuberculous mycobacteria (NTM), has gained prominence as a significant pathogen responsible for a wide range of infections, including both pulmonary and extrapulmonary manifestations.^1^ The increasing incidence of MAB infections, especially in individuals with underlying, lifelong lung conditions such as cystic fibrosis and COPD, underscores the critical need to understand the factors driving this pathogen's proliferation. Treating MAB infection is exceptionally challenging due to the high-level intrinsic resistance to most antibiotics commonly used for other bacterial infections, such as the majority of β-lactams and tetracyclines.^2–4^ Additionally, the antibiotics for anti-tuberculosis treatment, including first-line drugs (e.g. rifampicin and isoniazid), as well as some second-line agents (e.g. fluoroquinolones and capreomycin), are ineffective against this pathogen.^5^ Consequently, MAB has been dubbed an ‘antibiotic nightmare’.^5,6^ As a result, multidrug therapy for MAB infections typically leads to bacterial culture conversion in <50% of patients, while the remaining patients either remain culture-positive, experience relapse or ultimately face fatal outcomes.^7^

Among the available therapeutic options, macrolides have served as a cornerstone in the treatment regimen for MAB due to their ability to improve clinical outcomes and enhance cure rates when the bacterial strain is susceptible.^8,9^ However, the efficacy of macrolides is severely compromised by the pathogen’s resistance mechanisms, including both intrinsic resistance and acquired resistance. This review aims to provide an overview of the current knowledge on macrolide resistance in MAB by focusing on epidemiological trends, molecular mechanisms and the variability in predictive markers for resistance across different regions. We used the keywords ‘MAB, drug resistance, mutations, macrolide’ to search for research articles on PubMed on 15 June 2024, which returned 73 studies. We found that the percentage of macrolide-resistant strains explained by *erm(41)* or *rrl* mutations varied substantially across different studies, and we have discussed the potential causes of these discrepancies.

## Intrinsic, inducible resistance to macrolides

Macrolides work by binding to the 50S ribosomal subunit in bacteria, specifically binding to the nascent peptide exit tunnel.^10^ This blocks elongation of the nascent polypeptide chain by physically obstructing the path through which the growing protein exits the ribosome. By blocking the ribosome’s exit tunnel, macrolides prevent the addition of new amino acids to the growing polypeptide chain.^11,12^ This halts protein synthesis at the elongation stage, leading to incomplete or non-functional proteins.^13^ By stopping protein synthesis, macrolides exhibit bacteriostatic effects—inhibiting bacterial growth rather than killing the bacteria.^14,15^

**Figure 1.**
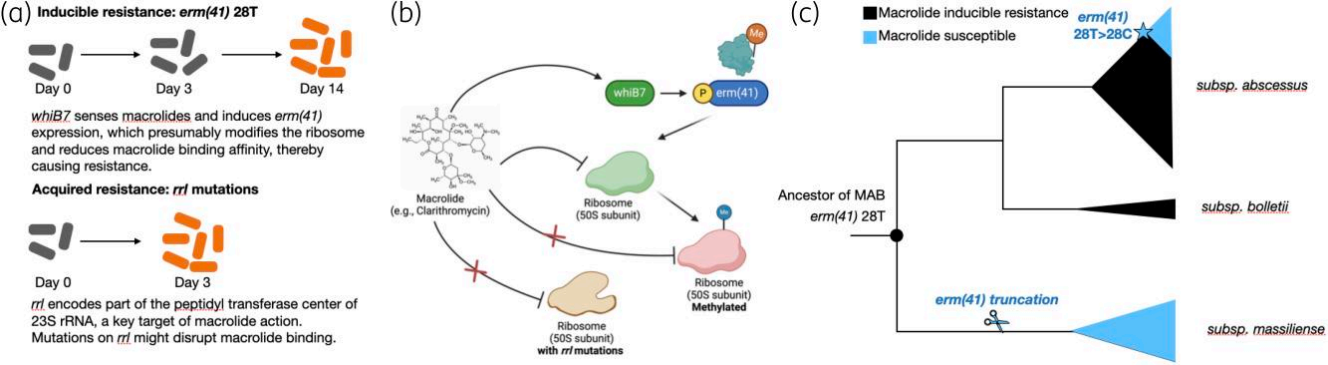
Mechanisms of macrolide resistance in *M. abscessus* and evolutionary changes in *erm(41)*. (a) Two primary mechanisms of macrolide resistance in MAB. Inducible resistance is mediated by *erm(41)* with a 28T allele, which is up-regulated in response to macrolides via *whiB7*, leading to ribosomal methylation and reduced drug binding. MAB remain susceptible at early time points (Day 3) but develop resistance later, typically measured by Day 14. Acquired resistance occurs through mutations in *rrl*, leading to macrolide resistance detected as early as Day 3. (b) Schematic representation of macrolide resistance mechanisms. Macrolides inhibit MAB growth by binding to the 50S ribosomal subunit. In *erm(41)*-mediated resistance, *whiB7* senses macrolides and activates *erm(41)* expression, leading to ribosomal methylation and reduced macrolide binding. In acquired resistance, *rrl* mutations disrupt macrolide binding to the ribosome, conferring resistance. (c) Phylogenetic reconstruction of macrolide resistance evolution in the MAB complex. The ancestral *erm(41)* 28 T allele, associated with inducible macrolide resistance (black), was retained in MAB *subsp. abscessus* and *subsp. bolletii*. A lineage-specific mutation (28T→28C) led to macrolide susceptibility. In MAB *subsp. massiliense*, *erm(41)* was truncated, resulting in loss of inducible resistance.

The intrinsic resistance mechanism to macrolides in MAB is the presence of a functional *erm(41)* gene.^16^  *Erm* genes encode ribosome methylases that specifically methylate an adenine residue (A2058 in *E. coli* numbering) within the peptidyl transferase center of the 23S rRNA.^17^ This methylation induces conformational changes in the ribosome, reducing the binding affinity of macrolides. However, resistance caused by *erm(41)* in MAB is inducible, as its transcription is positively regulated by WhiB7 upon exposure to macrolide treatment (Figure 1a). Inducible resistance is measured by MIC experiments as late as Day 14, as bacteria carrying a functional *erm(41)* gene only develop macrolide resistance after the induction of *erm(41)*. To exhibit inducible drug resistance to macrolides, MAB needs to have a functional version of *erm(41)*, characterized by the 28T genotype and without truncating or promoter mutations.^18^

Not all MAB strains carry the drug-resistant version of *erm(41)*, as the genotypes of the *erm(41)* gene vary across different subspecies of MAB. MAB comprises three subspecies: *subsp*. *abscessus*, *subsp*. *massiliense* and *subsp*. *bolletii*. The ancestor of subsp. *massiliense* had the *erm(41)* gene truncated due to a frame-shift deletion in the C terminal region, and therefore most strains of subsp. *massiliense* are intrinsically susceptible to macrolides.^19^ Infections caused by strains of MAB subsp. *massiliense* can be treated with macrolides and are associated with favorable treatment outcomes.^20^ However, the ancestors of *subsp*. *abscessus* and *subsp*. *bolletii* both carry the 28T genotype and a full-length version of *erm(41)*, conferring inducible-resistance to macrolides. Interestingly, at a sub-ancestor position in the phylogeny of subsp. *abscessus*, an early diverging clade accumulated a 28T > C mutation, which rendered strains from this clade susceptible to macrolides (Figure 1b).^21^ The two mutations resulting in inactivation of *erm(41)* occurred independently in subsp. *abscessus* and *subsp*. *massiliense*, suggesting a process of natural selection instead of genetic drift. It seems plausible that a functional *erm(41)* provides an advantage in the presence of macrolides but may impose a fitness cost in their absence, although definitive experimental evidence for this remains lacking. Therefore, the intrinsic macrolide susceptibility profiles of MAB subspecies are heterogeneous, with *subsp*. *massiliense* strains being drug-susceptible, *subsp*. *bolletii* being drug-resistant and *subsp*. *abscessus* further divided into two subpopulations: those carrying 28T are drug-resistant, while those carrying 28C are drug-susceptible.

## Distinct composition of MAB subspecies across continents

**Figure 2.**
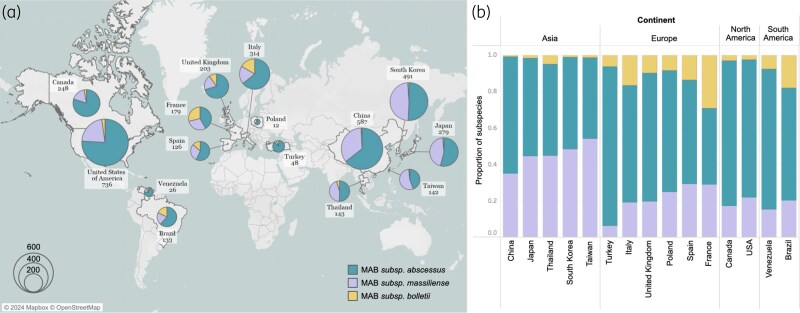
The relative proportions of the three *M. abscessus* subspecies across different geographic regions. (a) Pie charts illustrating the composition of *subsp. abscessus, subsp. massiliense* and *subsp. bolletii* in various countries/regions. (b) Bar plots displaying the relative proportions, highlighting the increased prevalence of *subsp. massiliense* in Asia and *subsp. bolletii* in Europe.

Given the variability in macrolide susceptibility between MAB subspecies, the identification of subspecies has the potential to infer drug susceptibility and guide treatment regimens.^19^ We summarized the composition of MAB subspecies reported from different countries (Table 1, Figure 2a). Out of 73 papers reporting subspecies information of their MAB isolates, 30 studies from 15 countries were included in this review based on the following criteria: (i) more than 10 isolates were analyzed from each study; and (ii) all three subspecies were genotyped. Across the 15 countries from which we obtained subspecies data, *subsp*. *abscessus* consistently emerged as the most prevalent subspecies, ranging from 37.3% to 92.9% (averaging 63.7%). However, we noticed a disparate distribution of *subsp*. *massiliense* strains across continents. Countries in East and South Asia exhibit a significantly higher prevalence of *subsp*. *massiliense* strains, while countries in the Americas and Europe show a much lower prevalence (median percentage, Asia: 43.7% versus Europe 21.4% or Americas 19.2%, *P* = 0.0007 and 0.0004 respectively). For example, countries in Asia such as Japan, Thailand and China typically have around 40% to 50% *subsp*. *massiliense*. In contrast, countries in the Americas (e.g. USA and Canada) and Europe (e.g. France and the UK) have only 15.4% to 30.0% *subsp*. *massiliense* (Figure 2b). The factors driving the differing distribution of *subsp. massiliense* are unclear, and phylogeographic analyses of *subsp. massiliense* strains from different continents could help elucidate the origin and global dispersal of this subspecies. The prevalence of *subsp. bolletii* is consistently low, averaging 8.3% globally, but is notably higher in European countries, ranging from 6.3% to 31.0% with an average of 27.7% (Figure 2b).

**Table 1.** A summary of the proportion of MAB subspecies by study and country

Country/Region	Year	Authors	*Subsp. abscessus* (%)	*Subsp. massiliense* (%)	*Subsp. bolletii* (%)
Japan	2018	Yoshida, *et al*.^22^	74 (51.0%)	69 (47.6%)	2 (1.4%)
Japan	2019	Aono, *et al*.^23^	48 (56.5%)	35 (41.2%)	2 (2.4%)
Japan	2021	Yoshida, *et al*.^24^	29 (59.2%)	20 (40.8)	0 (0.0%)
Japan and Taiwan	2022	Yoshida, *et al*.^25^	112 (50.9%)	105 (47.7%)	3 (1.4%)
Taiwan	2017	Lee, *et al*.^26^	28 (41.8%)	38 (56.7%)	1 (1.5%)
Taiwan	2022	Jong, *et al*.^27^	35 (46.7%)	39 (52.0%)	1 (1.3%)
China	2018	Zheng, *et al*.^28^	218 (56.6%)	163 (42.3%)	4 (1.0%)
China	2018	Li, *et al*.^29^	52 (92.9%)	4 (7.1%)	0 (0.0%)
China	2021	Guo, *et al*.^30^	32 (74.4%)	11 (25.6%)	0 (0.0%)
China	2022	Li, *et al*.^31^	76 (73.8%)	27 (26.2%)	0 (0.0%)
Korea	2014	Lee*, et al*.^32^	202 (50.0%)	199 (49.3%)	3 (0.7%)
Korea	2019	Huh, *et al.*^33^	47 (54.0%)	38 (43.7%)	2 (2.3%)
Thailand	2018	Ananta, *et al*.^34^	68	—	—
Thailand	2023	Sukmongkolchai, *et al*.^35^	72 (50.4%)	64 (44.8%)	7 (4.9%)
Switzerland	2021	Maurer, *et al*.^36^	29	—	—
Turkey	2023	Sürücüoğlu, *et al*.^37^	42 (87.5%)	3 (6.3%)	3 (6.3%)
France	2017	Mougari, *et al*.^38^	28 (52.8%)	12 (22.6%)	13 (24.5%)
France	2017	Mougari, *et al*.^39^	47 (37.3%)	40 (31.8%)	39 (31.0%)
United Kingdom	2018	Lipworth, *et al*.^40^	143 (70.4%)	40 (19.7%)	20 (9.9%)
Italy	2020	Teri, *et al*.^41^	202 (64.3%)	60 (19.1%)	52 (16.6%)
Spain	2015	Rubio, *et al.*^42^	11 (68.8%)	1 (6.3%)	4 (25.0%)
Spain	2023	Ruedas-López, *et a*l.^43^	50 (52. 1%)	33 (34.4%)	13 (13.5%)
Spain	2024	Buenestado-Serrano, *et al*.^44^	11 (78.6%)	3 (21.4%)	0 (0.0%)
Poland	2023	Kania, *et al*.^45^	8 (66.7%)	3 (25.0%)	1 (8.3%)
Canada	2016	Christianson, *et al*.^46^	104 (83.9%)	20 (16.1%)	0 (0.0%)
Canada	2020	Sharma, *et al*.^47^	94 (75.8%)	23 (18.6%)	7 (5.7%)
United States of America	2021	Davidson, *et al.*^48^	440 (78.9%)	107 (19.2%)	11 (2.0%)
United States of America	2021	Realegeno, *et al*.^49^	47 (69.1%)	20 (29.4%)	1 (1.5%)
United States of America	2022	Shallom, *et al*.^50^	72 (65.5%)	33 (30.0%)	5 (4.6%)
Venezuela	2015	Ramírez, *et al.*^51^	20 (77.0%)	4 (15.4%)	2 (7.7%)
Brazil	2018	de Carvalho, *et al*.^52^	82 (61.7%)	27 (20.3%)	24 (18.1%)

## Acquired resistance to macrolides

To date, three acquired drug resistance mechanisms have been characterized for macrolide resistance in MAB. First, the most common path is through mutations in the *rrl* gene,^36^ which encodes part of the peptidyl transferase center of 23S rRNA, a key target of macrolides. Mutations in *rrl*, particularly at positions 2058 and 2059, can change the conformation of the ribosome, which disrupts macrolide binding, allowing bacterial survival despite the presence of typically inhibitory antibiotic concentrations.^53,54^ All three subspecies of MAB can accumulate *rrl* mutations in clinical strains (Table 2). Unlike inducible resistance, acquired resistance due to *rrl* mutations does not require regulation by WhiB7, and drug-resistance profiles in MIC experiments can be measured as early as Day 3 (Figure 1a). Second, strains from the 28C clade of *subsp. abscessus* can acquire mutations that revert *erm(41)* 28C back to *erm(41)* 28T, resulting in a functional *erm(41)* and again causing inducible resistance.^21^ This is evident in a recent study that analyzed ∼5000 MAB isolates’ genomes and found that the revertant mutations are common in the clade with the 28C genotype. Third, it has been found that strains of *subsp. massiliense* can acquire an intact and functional version of *erm(41)*, presumably through horizontal gene transfer (HGT), leading to acquired inducible resistance.^21^ For example, two *subsp. massiliense* strains with full-length *erm(41)* gene sequences and inducible macrolide resistance were found in a group of 43 strains collected at NIH.^54^ This HGT mechanism is newly reported, and its frequency is yet to be characterized.

**Table 2.** A summary of genotypic and phenotypic resistance to macrolides across countries

Country/Region	Year	Authors	Subspecies/*erm(41)* genotypes		Number of Isolates	Number of *rrl* mutants	Number of resistant mutants on Day 3^a^	Number of resistant mutants on Day 14^b^	Day 3 resistance explained by *rrl* mutations
Japan	2018	Yoshida, *et al*.^22^	ABS	*erm(41)* 28T	66	1	7	58	1/7
				*erm(41)* 28C	8	0			
			MAS		69	2	2	2	2/2
			BOL		2	2	2	2	2/2
Japan	2021	Yoshida*, et al.*^24^	ABS	*erm(41)* 28T	24	0	6	23	0/6
				*erm(41)* 28C	5	0	0	0	0/0
			MAS		20	2	4	4	2/4
Taiwan	2017	Lee, *et al*.^26^	ABS	*erm(41)* 28T	18	0	3	16	0/3
				*erm(41)* 28C	10	0			
			MAS		38	2	2	2	2/2
			BOL		1	0	0	1	—
Taiwan	2022	Jong*, et al.*^27^	ABS	*erm(41)* 28T	24	0	0	22	0/0
				*erm(41)* 28C	11	0			
			MAS		39	1	1	1	1/1
			BOL		1	—	—	—	—
China	2018	Zheng, *et al.*^28^	ABS	*erm(41)* 28T	185	7	11	182	7/11
				*erm(41)* 28C	33	3	3	3	3/3
			MAS		163	10	17	19	10/17
			BOL		4	—	—	—	—
China	2018	Li, *et al*.^29^	ABS	*erm(41)* 28T	34	3	3	34	3/3
				*erm(41)* 28C	9	0	0	0	0/0
			MAS		4	1	—	—	—
China	2021	Guo, *et al.*^30^	ABS	*erm(41)* 28T	22	0	4	22	0/4
				*erm(41)* 28C	10	0			
			MAS		11	0	2	2	0/2
			BOL		0	0	—	—	—
China	2022	Li, *et al.*^31^	ABS	*erm(41)* 28T	44	1	1	41	1/1
				*erm(41)* 28C	32				
			MAS		27	1	1	0	1/1
Korea	2014	Lee, *et al*.^32^	ABS	*erm(41)* 28T	202	2	48	120	2/48
				*erm(41)* 28C					
			MAS		199	4	15	0	4/15
			BOL		3	0	1	2	0/1
Thailand	2023	Sukmongkolchai, *et al.*^35^	ABS	*erm(41)* 28T	54	20	17	51	20/20
				*erm(41)* 28C	18		3	3	
			MAS		64	0	0	0	0/0
			BOL		7	0	0	7	0/0
Poland	2023	Kania, *et al.*^45^	ABS	*erm(41)* 28T	6	0	—	6	—
				*erm(41)* 28C	1	0	—	0	—
			MAS		3	0	—	1	—
			BOL		1	0	—	1	—
Canada	2020	Sharma, *et al.*^47^	ABS	*erm(41)* 28T	70	1	2	70	1/2
				*erm(41)* 28C	24	0		24	
			MAS		23	1	1	1	1/1
			BOL		7	0	0	5	0/0
United States of America	2021	Realegeno, *et al*.^49^	ABS	*erm(41)* 28T	55	0	—	55	—
				*erm(41)* 28C	21	0	—	21	—
			MAS		27	2	—	2	—
			BOL		1	0	—	1	—
Venezuela	2015	Ramírez, *et al*.^51^	ABS	*erm(41)* 28T	7	0	—	7	—
				*erm(41)* 28C	13	0	—	0	—
			MAS		4	0	—	0	—
			BOL		2	0	—	2	—
Brazil	2018	de Carvalho, *et al.*^52^	ABS	*erm(41)* 28T	73	1	1	73	1/1
				*erm(41)* 28C	6	0	—	0	—
			MAS		27	0	—	0	—
			BOL		24	0	4	—	0/4

^a^Indicates acquired resistance.

^b^Indicates inducible resistance.

It is noteworthy that MAB *subsp. abscessus* and *subsp. bolletii* strains carrying the *erm(41)* 28T variant can further accumulate *rrl* mutations. This additional accumulation of *rrl* mutations suggests that while the *erm(41)* 28T confers inducible resistance, the clinical use of macrolides continues to apply selective pressure on MAB, potentially driving further evolution and increasing fitness during the ‘inducible’ resistance window. Alternatively, it is possible that the level of resistance conferred by *erm(41)* 28T alone is not sufficient to fully evade killing by macrolides, as drug concentrations may exceed the MIC observed in *erm(41)* 28T variants. Distinguishing between these two possibilities could provide insights into optimising macrolide use in treating MAB infections.

While direct transmission of MAB between humans is considered rare,^55,56^ drug-resistant strains could still propagate and infect others through environmental intermediaries.^57^ Therefore, the prevalence of acquired drug resistance mutations in clinical isolates can provide valuable insights into their spread and persistence within populations. We observed that the prevalence of *rrl* mutants vary across different studies, even within the same country (Table 2). For example, one study from China reported that only 2 out of 103 isolates (1.9%) carried *rrl* mutations and acquired resistance to clarithromycin.^31^ In contrast, other studies from China reported the prevalence of *rrl* mutations in MAB isolates ranges from 6.4% to 14.0%. The differences in the prevalence of *rrl* mutants across studies can be attributed to variations in patient populations, sampling biases, and local antibiotic use practices. Additionally, the potential impact of temporal factors and the clonal spread of drug-resistant MAB strains warrants further investigation.

## Can *erm(41)* and *rrl* mutations effectively predict phenotypic resistance to macrolides?

**Figure 3.**
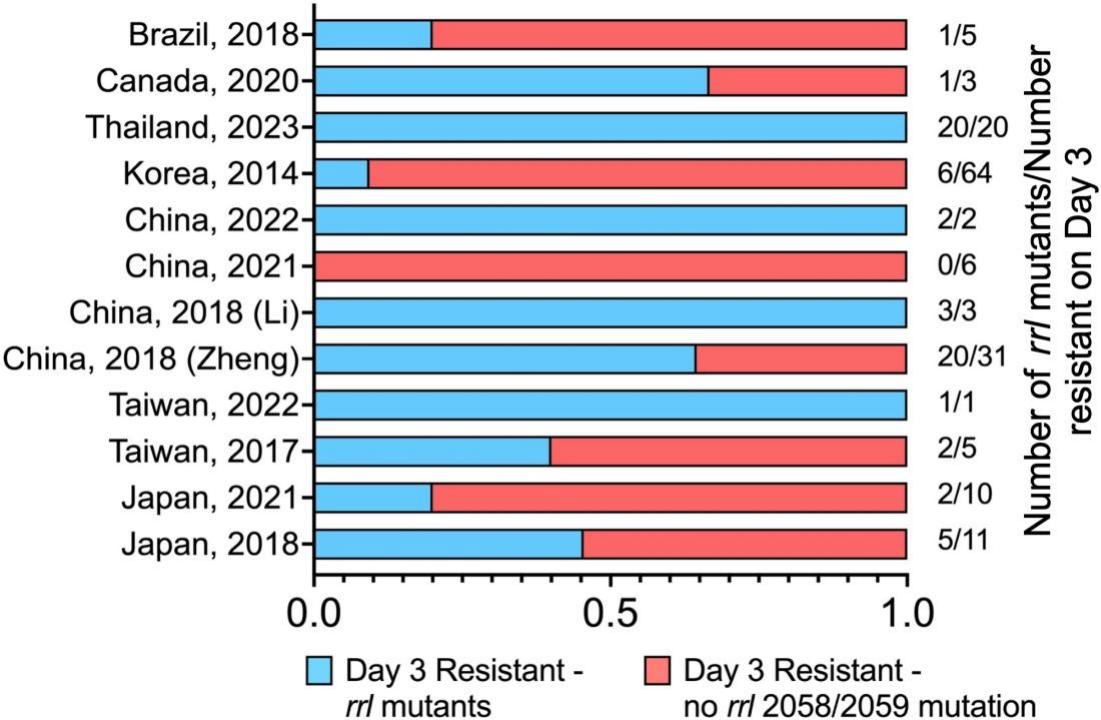
Sensitivity of rrl mutations in predicting macrolide resistance on Day 3 across different studies and geographic regions. A stacked bar chart showing the proportion of macrolide-resistant isolates on Day 3 that harbor rrl mutations versus those without rrl mutations. Each bar represents a study or dataset, with the total number of resistant isolates on Day 3 indicated on the right. The proportion of rrl mutants varies across studies, with some datasets (e.g. Thailand 2023 and China 2022) showing complete correlation between rrl mutations and resistance, while others (e.g. China 2021 and Brazil 2018) exhibit a lower association. These findings highlight variability in the predictive value of rrl mutations for early macrolide resistance.

Overall, the presence of a functional version of *erm(41)* is a strong indicator of inducible resistance to macrolides (Table 2). For instance, a study from South Korea analyzed 404 MAB isolates and found that all 122 cases of inducible resistance to macrolides can be explained by the *erm(41)* 28T variant.^32^ Similarly, a study from Thailand analyzed 143 MAB isolates, of which 58 exhibited inducible resistance, all associated with the *erm(41)* 28T genotype.^31^ A study from China also reported high sensitivity, with 40 out of 44 isolates with inducible resistance explained by *erm(41)* 28T.^31^ However, the sensitivity of *rrl* mutations in predicting acquired macrolide resistance varies across studies (Figure 3). For example, in a study from South Korea, among 64 isolates with acquired resistance, only 6 (9.3%) carried *rrl* mutations.^32^ Similarly, in a Polish study, only 1 of 8 MAB *subsp. abscessus* isolates with acquired resistance had *rrl* mutations (12.5%).^45^ In contrast, a Thai study found that all 20 isolates (100%) with acquired macrolide resistance had *rrl* mutations,^35^ whereas another study from Thailand reported that *rrl* mutations accounted for only 7 of 16 acquired-resistant strains (43.8%).^34^ These inconsistencies highlight the complexity of using *rrl* mutations to predict acquired macrolide resistance. Further, this variation likely suggests that there are additional resistance mechanisms which have not been identified and characterized in MAB.

The specificity of using *erm(41)* 28T to predict inducible resistance and *rrl* mutations to predict acquired resistance is generally close to 100% (Table 2), with a few exceptions in studies with a small number of isolates. For example, in the aforementioned Thailand study, 3 out of 54 *subsp. abscessus* isolates with the *erm(41)* 28T genotype were drug-susceptible (Specificity: 94.4%).^35^ Similarly, a Chinese study identified 3 macrolide-susceptible isolates out of 185 *erm(41)* 28T-positive isolates (Specificity: 99.0%).^28^ A Polish study also reported MAB isolates with both the *erm(41)* 28T genotype and *rrl* mutations (in the 2058-2059 region) that remained susceptible to clarithromycin.^45^ Because of the high specificity of *erm(41)* 28T and *rrl* mutations in predicting macrolide resistance, some clinical microbiology labs now perform PCR-based tests for *erm(41)* and *rrl* genotypes to provide rapid diagnostic results to clinicians.

## Potential causes of the discrepancies of predictive sensitivity

Three major causes for the discrepancies in predictive sensitivity have been considered:

1. Uncharacterized Mutational Sites in Known Drug-resistant Genes. Currently, only canonical mutations in *erm(41)* and *rrl* were included for analysis. However, new mutations from these genes could confer drug resistance. For example, a recent study conducted an *in vitro* screen of macrolide resistance in MAB and have identified new variants of *rrl* that can cause drug resistance to macrolide.^58^ They screened 71 macrolide-resistant mutants based on a clinical strain and found only 39 (54.9%) had mutations at 2270/2271 (refers to 2058 and 2059 in *E. coli*) while 32 (45.1%) did not. For those 32 mutants without 2270/2271 mutations, 21 (29.6% of total) had mutations at other *rrl* sites, including 2057/2058/2059. They also conducted this experiment in reference strain ATCC 19977 and found only 20 out of 62 mutants (32.3%) had mutations at 2270/2271, while 40 (64.5%) had other *rrl* mutations. This suggests a broader spectrum of drug-resistant mutational sites in *rrl* than previously recognized.
2. Experimental Variability. A study by Lipworth *et al*.^40^ used mutations in the *erm(41)* and *rrl* genes to predict clarithromycin resistance in a collection of 203 clinical isolates from the UK.^40^ They reported that the sensitivity of using known mutations to predict resistance was high (∼95%), but the specificity was low (66%), as 27 of 101 isolates predicted to have inducible resistance turned out to be susceptible in experiments. However, they repeated their study with another collection of 259 isolates and found that the sensitivity was similarly high (98%, 194/197) while the specificity increased to 98% (61/62).^59^ As discussed by the authors, this improved performance of prediction may reflect the increased experience gained in the experimental method by laboratory staff over time, implying that the previous low specificity was due to experimental incongruence.^59^
3. Uncharacterized Drug-Resistance Genes. Other genes that have been associated with macrolide resistance in MAB include ribosome protection factors, novel methyltransferases and efflux pumps.^54,60,61^ The accessory genome of MAB is vast, having expanded through HGT^62^ and a potential source of genes encoding these, and other, novel antibiotic resistance mechanisms. Despite the potential importance of the MAB accessory genome, it has not been thoroughly analyzed to evaluate its role in macrolide resistance. Understanding this aspect requires systematic efforts, such as whole-genome sequencing of clinical isolates with unexplained macrolide resistance to identify novel resistance genes. Comparative genomic studies can identify genes that are enriched in resistant strains, while further functional assays, such as gene knockout and overexpression, are essential to validate their specific contributions to resistance mechanisms.

## Future directions and concluding remarks

### Wide MIC spectrums suggest unknown determinants

An interesting observation is that even among strains with the same *erm(41)* T28 genotype and no *rrl* mutations, their MICs for macrolides on Day 3 can differ by as much as 30-fold, ranging from 0.06 to 2 mg/L.^28^ Similarly, strains with the *erm(41)* C28 genotype and no *rrl* mutations also exhibit an MIC range that spans 10-fold.^31^ These patterns have been consistently observed across various studies, suggesting that additional factors influence macrolide MIC levels despite the shared *erm(41)* genotype. Possible explanations for this observation include the complexity of regulatory networks controlling *erm(41)* expression,^63^ and modulating factors such as efflux pumps.^64^

### Data from Africa is lacking

Currently, no studies from Africa have reported characterization of MAB isolates from clinical settings. The absence of data on MAB in Africa likely indicates the lack of healthcare infrastructure to diagnose human MAB infections and conduct surveillance to collect clinical MAB isolates. However, a recent study sampling MAB isolates from wildlife in Africa showed a high ratio of *subsp. bolletii*, accounting for 37 of the 102 MAB isolates.^65^ Understanding the environmental niches from which MAB can be isolated may provide broader insights into its evolutionary history. However, the limited human data on MAB isolates in Africa restricts our understanding of the epidemiology and clinical impact of these infections in human populations.

### Variations in *erm(41)* induction

Because inducible expression of *erm(41)* is controlled by *whiB7*, it was suggested that different macrolides may variably induce *erm(41)* expression, leading to differences in resistance phenotypes. For example, a study published in 2012 found that azithromycin induced lower *erm(41)* expression levels than clarithromycin, suggesting azithromycin could be the preferred treatment.^66,67^ However, a 2020 study challenged this observation, reporting that macrolide resistance was induced more rapidly in various MAB isolates upon exposure to azithromycin than to clarithromycin^63^ Notably, these studies employed different methodologies: the 2012 study used RT-PCR to measure *erm(41)* expression, whereas the 2020 study utilized a β-galactosidase activity reporter system under the *erm(41)* promoter.^63,66^ Additionally, they tested different MAB strains,^63,66^ raising the possibility that the observed discrepancies may stem from methodological differences or strain-specific genetic backgrounds. Therefore, further research is warranted to further clarify the variation in *erm(41)* induction across different macrolides and representative clinical isolates of MAB.

### The future of overcoming macrolide resistance

Several promising compounds have been shown to combat macrolide-inducible resistance in MAB, addressing the urgent need for more effective therapies against this challenging pathogen. For example, a recent study demonstrated that cyclophostin and cyclipostins analogues can counteract macrolide-induced resistance mediated by *erm(41)* in MAB, thereby restoring macrolide susceptibility.^68^ Particularly, Isoegomaketone (iEMK) demonstrates potential in mitigating macrolide-inducible resistance and effectiveness as a combination of clarithromycin and iEMK can reduce or prevent inducible resistance following 14 days of exposure.^69^ Additionally, the combination of rifaximin and clarithromycin has also shown promise in potentiating clarithromycin’s activity, with synergistic effects observed *in vitro* and in infection models.^70^ MRX-5, an oral oxaborole prodrug, has shown significant efficacy against various MAB isolates, including drug-resistant strains, by targeting leucyl-tRNA synthetase.^71,72^ Enhanced spectinomycin derivatives are being developed to overcome efflux-mediated resistance and have shown up to 64-fold increased potency against MAB over spectinomycin.^73^ Additionally, SPR720, an aminobenzimidazole bacterial DNA gyrase inhibitor, is undergoing clinical trials and could potentially become the first novel NTM antibiotic approved for use.^74,75^ These diverse therapeutic approaches mark significant advancements in the fight against MAB infections, offering hope for overcoming macrolide resistance and achieving better treatment outcomes in the future.

In conclusion, this review highlights the intricate and multifaceted nature of macrolide resistance in MAB. While *erm(41)* and *rrl* mutations are key contributors to resistance, the variability of using these mutations in predicting phenotypic drug resistance underscores the limitations of molecular diagnostic strategies, which rely on genotypic markers that may not fully capture the resistance phenotype. Moreover, the variability in subspecies distribution and resistance patterns points to the need for region-specific studies and tailored therapeutic approaches. Addressing these knowledge gaps is essential for improving clinical outcomes and guiding future research in combating MAB infections.
